# Immuno-enhancement effects of *Platycodon grandiflorum* extracts in splenocytes and a cyclophosphamide-induced immunosuppressed rat model

**DOI:** 10.1186/s12906-019-2724-0

**Published:** 2019-11-21

**Authors:** Eun-Mi Noh, Jeong-Mi Kim, Hak Yong Lee, Hyun-Kyung Song, Sang Ok Joung, Hye Jeong Yang, Min Jung Kim, Kang Sung Kim, Young-Rae Lee

**Affiliations:** 1 Department of Oral Biochemistry, and Institute of Biomaterial-Implant, College of Dentistry, Wonkwang University, 460, Iksandae-ro, Iksan, Jeonbuk 54538 Republic of Korea; 2 INVIVO Co. Ltd., 310, GVSC, Iksandae-ro, Iksan, Jeonbuk 54538 Republic of Korea; 3 JIRISANDONGUICHON Co. Ltd, 2000-89 Sincha-ro, Chahwang-myeon, Sancheong, Gyeongnam 52206 Republic of Korea; 4 Korea Food Research Institute, 245, Nongsaengmyeong-ro, Iseo-myeon, Wanju-gun, Jeollabuk-do 55365 Republic of Korea; 5 Department of Food Science and Nutrition, Yong In University, Yongin, Gyeonggi-do 17092 Republic of Korea

**Keywords:** *Platycodon grandiflorum*, Immunostimulation, Cyclophosphamide, Cytokine, Immune cells

## Abstract

**Background:**

*Platycodon grandiflorum* is a flowering plant that is used in traditional medicine for treating pulmonary and respiratory disorders. It exerts various pharmacological effects, including immunomodulatory and anti-cancer activities. The purpose of this study was to confirm the in vitro and in vivo immune-enhancing effects of *P. grandiflorum* extract (PGE) on splenocytes isolated from cyclophosphamide (CP)-induced immunosuppressed rats.

**Methods:**

For in vitro analysis, splenocytes were treated with PGE at various doses along with CP. Cell viability was measured by a WST-1 assay, and NK cell activity and cytotoxic T lymphocyte (CTL) activity was also examined. In addition, immunoglobulin A (IgA), IgG, and cytokine levels were measured. For in vivo analysis, Sprague Dawley rats were treated with various doses of PGE along with CP. Complete blood count (CBC) was performed, and plasma levels of IgA, IgG, TNF-α, IFN-γ, IL-2, and IL-12 were quantified. Additionally, tissue damage was assessed through histological analyses of the thymus and spleen.

**Results:**

PGE treatment enhanced cell viability and natural killer cell and cytotoxic T lymphocyte activity, and increased the production of CP-induced inflammatory cytokines (TNF-α, IFN-γ, IL-2, and IL-12) and immunoglobulins (IgG and IgA) in splenocytes. In addition, in CP-treated rats, PGE treatment induced the recovery of white blood cell, neutrophil, and lymphocyte counts, along with mid-range absolute counts, and increased the serum levels of inflammatory cytokines (TNF-α, IFN-γ, IL-2, and IL-12) and immunoglobulins (IgG and IgA). Moreover, PGE attenuated CP-induced spleen and thymic damage.

**Conclusions:**

Our results confirmed that PGE exerts an immune-enhancing effect both in vitro and in vivo, suggesting that PGE may have applications as a component of immunostimulatory agents or as an ingredient in functional foods.

## Background

*Platycodon grandiflorum* (PG) roots have traditionally been used as a herbal medicine and food for the treatment of various pulmonary diseases and respiratory disorders in China, Japan, and Korea [1]. PG has been found to contain numerous chemical components, including saponins, polysaccharides, flavonoids, phenolic acids, fatty acids, and sterols [2]. In addition, PG performs various pharmacological activities that have anti-diabetic [3], anti-cancer [4], anti-inflammatory [5], immunomodulatory [6], and anti-allergic [7] effects.

Organs of the immune system, such as the bone marrow, thymus, lymph nodes, and spleen, play a major role in mediating the immune response and to ensure that a protective response to negate the effects of harmful stimuli is established [8]. These highly sophisticated and complex biological responses involve macrophages, dendritic cells, and various immune cells, including lymphocytes [9]. Lymphocytes, which comprise white blood cells, can be divided into T cells, B cells, and natural killer cells (NK). NK cells belong to the innate immune system and play a major role in defending the host from cancer cells, bacteria, and virus-infected cells [10]. T cells and secreted cytokines are associated with adaptive or cell-mediated immune responses, whereas B cells and antibodies are key factors in the humoral immune response [11]. Further, the imbalances in the immune system are associated with autoimmune diseases and disorders caused by inflammatory responses in various organs [12].

Cyclophosphamide (CP), a commonly used alkylating agent for chemotherapy, exerts myelosuppressive, immunosuppressive, and cytotoxic effects [13, 14]. Administration of CP can lead to a sudden change in Th1/Th2 bias, resulting in immunosuppression. In previous studies, the immunological effects of CP were found to decrease the proliferation of T cells along with the levels of secreted Th1 cytokines (tumor necrosis factor [TNF]-α, interferon [IFN]-γ, interleukin [IL]-2, and IL-12) and Th2 cells (IL-4, IL-6, and IL-10) [15, 16].

In order to develop potential immune-enhancing agents that can be used as components of functional foods, increasing attention is being paid to the immunomodulatory properties of plants with a broad spectrum of therapeutic properties, as plant-based therapeutic agents are associated with relatively low toxicities [17, 18]. Although a recent report indicated that PG exerts an anti-tumor effect by improving the immune function [19], to our knowledge, no data are available regarding its immune-enhancing effects in a CP-induced immunosuppression model. Therefore, in the present study, we confirmed the in vitro and in vivo immune-enhancing effects of PG extract (PGE) on splenocytes isolated from immunosuppressed rats generated by CP administration.

## Methods

### Preparation of PGE

PG was manufactured by JIRISANDONGUICHON Co. Ltd. (Sancheong-Gun, Gyeongnam, Korea). It was formally identified by the Rural Development Administration of the Korean Government. The dried roots of PG were purchased from JIRISANDONGUICHON, and a voucher specimen (no. 180426) was deposited at INVIVO Co. Ltd., Iksan, Korea. The extract was prepared as follows: briefly, the dried roots (100 g) were extracted in an electric boiling pot for 3 h with 1000 mL of distilled water at 100 °C, and filtered using a 0.45-μm syringe filter. The extract solution was evaporated under 40 mmHg using a rotary evaporator (N-1110S-W; Eyela, Tokyo, Japan), and was then used for each experiment.

The main components present in PGE were analyzed using an HPLC system (Shimadzu LC-30 Series, Shimadzu Corporation, Nakagyo-ku, Kyoto, Japan) with an SHISEIDO Capcell Pak C18 column (3.0 mm i.d. × 50 mm, 3 μm). Signals were detected using a MS system (Shimadzu LCMS-8030, Shimadzu Corporation, Nakagyo-ku, Kyoto, Japan). The levels of Platycodin D in PGE were quantitated using a calibration curve established by injecting dilutions of each Platycodin D standard (1–10 μg/mL) into the HPLC system (correlation coefficient ≥ 0.996). Standards (Platycodin D) for HPLC analysis were obtained from Sigma-Aldrich (St Louis, MO, USA).

### Animals

Animal experiments were performed as previously described [20]. Sprague Dawley (SD) rats were purchased from Samtaco Inc. (Osan, Gyeonggi-do, Korea), and acclimated to the following environmental conditions for 7 days: 12-h light/12-h dark cycle; temperature, 23 ± 1 °C; humidity, 50 ± 5%; and illumination, 150–300 lx. Rats were randomly assigned to five groups (10 rats per group). The protocols used for these animal studies were approved by the Committee on Care and Use of Laboratory Animals of Wonkwang University (Iksan, Jeollabuk-do, Korea; approval no. WKU18–17).

### Cell culture

Cells were cultured as previously described [20]. Briefly, the spleen of an 8-week-old SD rat was aseptically dissected to obtain splenocytes. The spleen was gently pressed with forceps and then forced through a 70-μm cell strainer (SPL Life Sciences, Pocheon-si, Gyeonggi-do, Korea). Cells were treated with red blood cell lysis buffer (Sigma-Aldrich, St. Louis, MO, U.S.A.). Isolated splenocytes were incubated in RPMI-1640 containing 10% fetal bovine serum (FBS) and 1% antibiotics (penicillin, streptomycin) (Invitrogen) in a 5% CO_2_ incubator.

### Cell viability

Cell viability were performed as previously described [20], using a WST-1 Assay Kit (ITSBio, Seoul, Gyeonggi-do, Korea) according to the manufacturer’s instructions. Briefly, splenocytes (2 × 10^5^ cells/well) were seeded into 96-well plates and then treated with PGE at various doses along with CP (1600 μg/mL), and incubated for 24 h in an incubator with 5% CO_2_ atmosphere. Cell viability was assessed using a WST-1 Assay Kit and a microplate plate reader (Tecan, Männedorf, Switzerland).

### NK cell activity assay

NK cell activity was measured as previously described [20]. Briefly, AR42J rat pancreas tumor cells obtained from the American Type Culture Collection (ATCC, Manassas, Va., U.S.A.) AR42J cells were used as target cells for the NK cell activity assay, and splenocytes were used as effector cells. Splenocytes were co-cultured with AR42J cells in 96-well plates at a 25:1 ratio of effector cells to target cells. The viability of AR42J cells was assessed using a CytoTox detection kit (TaKaRa, Shiga, Japan) and a microplate plate reader. NK cell activity was calculated as the viability of AR42J cells compared to control cells.

### Cytotoxic T lymphocyte (CTL) activity assay

CTL activity was determined as previously described [20]. Briefly, CTL activity was analyzed using the CytoTox detection kit (TaKaRa) according to the manufacturer’s protocol. Tumor cells (HL-60) obtained from ATCC were used as target cells for the CTL assay, and an effector cell to target cell ratio of 25:1 was used. CTL activity was calculated as the survival rate of HL-60 cells compared to that of control cells.

### Measurement of immunoglobulin a (IgA), IgG, and cytokine levels in splenocytes

Immunoglobulin (Ig) and cytokine levels were measured as previously described [20]. Briefly, splenocytes (2 × 10^5^ cells/well) were seeded into 96-well plates, after which cells were treated with various concentrations of PGE (0, 10, 30, 50, 100, 300, or 500 μg/mL) and CP (1600 μg/mL), and incubated for 24 h. The levels of IgA and IgG were measured using an immunoglobulin ELISA kit (AFFymetrix, Santa Clara, Ca., U.S.A and Abcam, Cambridge, U.K.) according to the manufacturer’s instructions. The levels of TNF-α, IFN-γ, IL-2, and IL-12 in the culture medium measured using Cytokine Activation Analysis Kits (R&D Systems, Minneapolis, MN, U.S.A.), according to the manufacturer’s instructions. The immunoglobulin and cytokine levels were measured using an ELISA reader.

### Complete blood count (CBC) and IgA, IgG, and cytokine analyses

These analyses were conducted as previously described [20]. Briefly, SD rats were orally administered PGE (0, 10, 30, or 100 mg/kg/day) and CP (5 mg/kg, once per day) for 28 days. After the final drug administration, rats were weighed and anesthetized with 5% isoflurane. Whole blood was collected. Next, the rats were euthanized by exsanguination. The numbers of white blood cells (WBCs), lymphocytes, neutrophils, and mid-range absolute counts (MID) were measured using a Hemavet 950 system (Drew Scientific Group, Dallas, Tx., U.S.A.). The levels of IgA, IgG, TNF-α, IFN-γ, IL-2, and IL-12 in the plasma were quantified using ELISA kits, according to the manufacturer’s instructions.

### Thymus and spleen histochemical analyses

Histochemical analyses of the thymus and spleen were performed as previously described [20]. Thymus and spleen tissues were fixed in 10% neutral buffered formalin. The organs were then processed for dehydrating with alcohol and embedding in paraffin. Samples were cut to 4-μm-thick section and stained with hematoxylin and eosin. Tissue damage was evaluated under a microscope.

### Statistical analysis

All experimental data are presented as mean ± standard error (SE), calculated using the statistical program SPSS version 12.0 (SPSS Inc., Chicago, IL, U.S.A). Results were analyzed by a one-way analysis of variance (ANOVA) and Duncan’s multiple range tests using SAS software (version 9.3; SAS Institute Inc., Cary, NC., U.S.A.). *P*-values < 0.05 were considered statistically significant.

## Results

### HPLC analysis of PGE

**Fig. 1:**
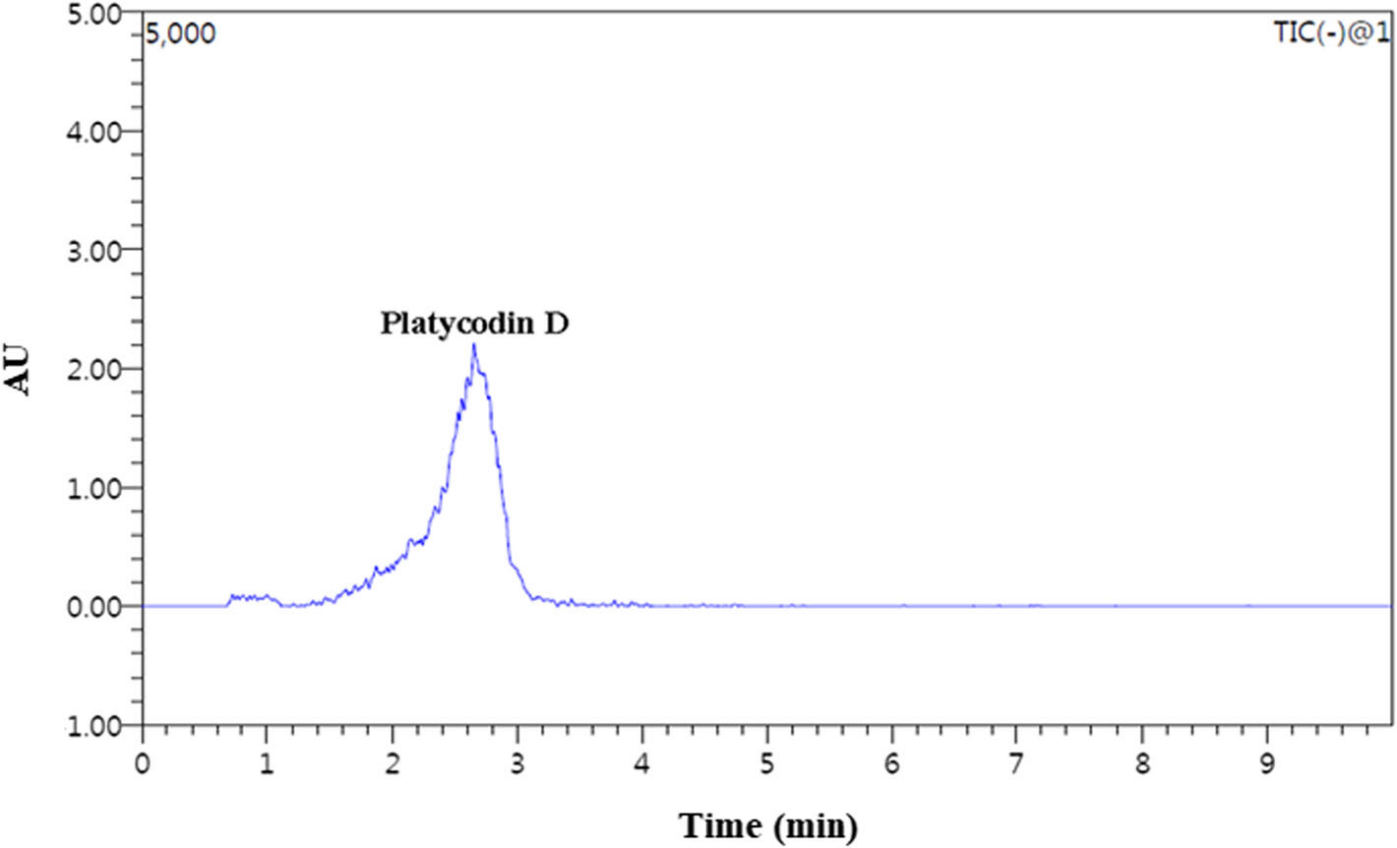
Representative high-performance liquid chromatography profile of PGE. Platycodin D was detected by LC-MS/MS

The physiological activity of *Platycodon grandiflorum* is attributed to the active compound, platycodin D. We performed LC-MS/MS analysis of the PGE, and found that PGE contained primarily platycodin D at a concentration of 22.6 mg/L (Fig. 1).

### Effect of PGE on CP-induced splenocyte viability

**Fig. 2:**
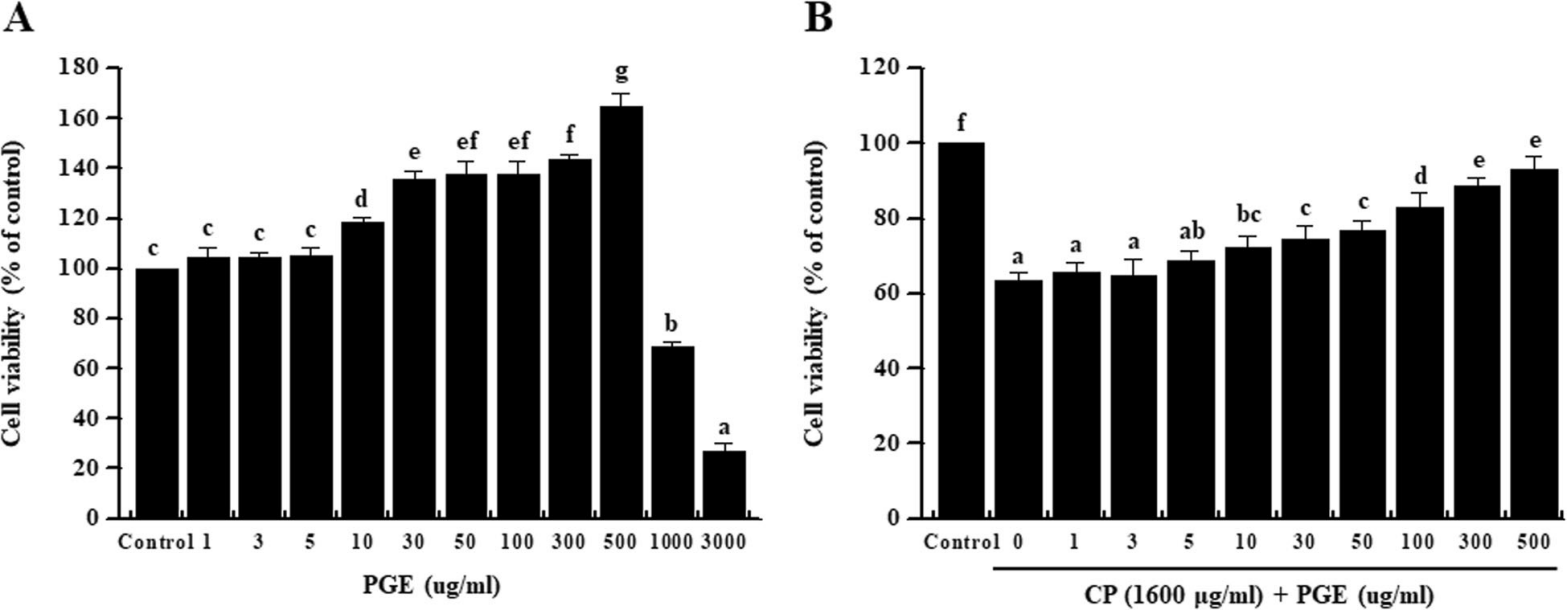
Effect of PGE on cytotoxicity of splenocytes and cell viability of CP-treated splenocytes. **a** Splenocytes were seeded into a 96-well plate with PGE (0, 1, 3, 5, 10, 30, 50, 100, 300, 500, 1000, and 3000 μg/mL). **b** Splenocytes were treated with PGE (0, 1, 3, 5, 10, 30, 50, 100, 300, and 500 μg/mL) and/or CP (1600 μg/mL). Cells were incubated for 24 h in under 5% CO_2_, and their viability was measured using a WST-1 Assay Kit. Bars labeled with different superscripts indicate significant differences (*P* < 0.05). Data are presented as the means ± standard errors (*n* = 3)

To confirm the cytotoxicity of PGE against splenocytes, cells were incubated with various concentrations of PGE (0, 1, 3, 5, 10, 30, 50, 100, 300, 500, 1000, or 3000 μg/mL) for 24 h. Cell viability was not effected at PGE concentrations < 500 μg/mL (Fig. 2a). Therefore, 500 μg/mL was considered the optimal non-toxic concentration, and subsequent in vitro experiments were performed at a PGE concentration of < 500 μg/mL. To investigate splenocyte viability, cells were incubated with CP (1600 μg/mL) and PGE (0, 1, 3, 5, 10, 30, 50, 100, 300, or 500 μg/mL) for 24 h. We found that PGE increased cell viability in a dose-dependent manner (Fig. 2b), indicating that PGE mitigates the reduction in cell proliferation caused by CP-induced immunosuppression.

### Effect of PGE on cytokine expression in CP-treated splenocytes

**Fig. 3:**
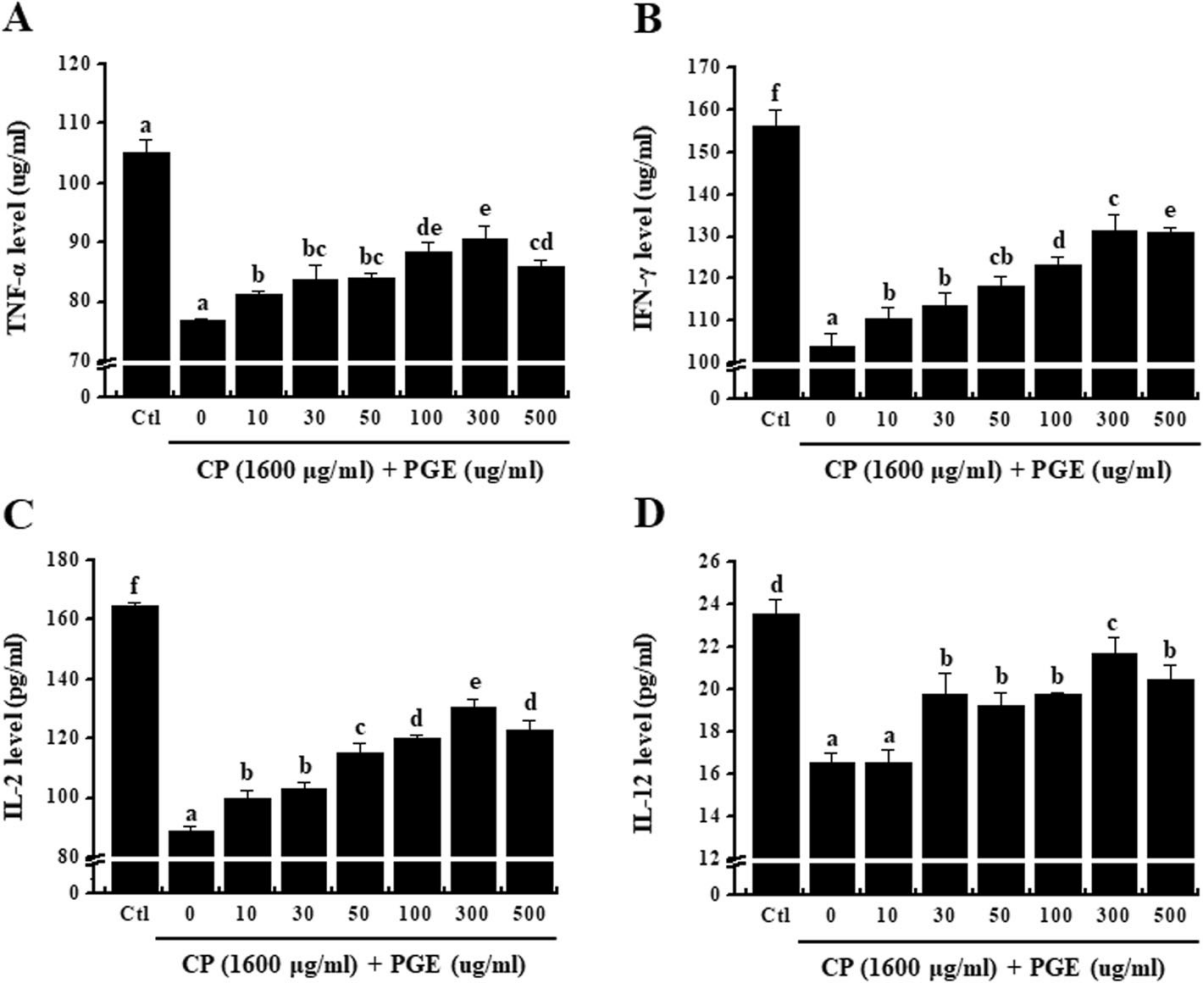
Effect of PGE on cytokine levels in CP-treated splenocytes. Cells were seeded into 96-well plates, followed by treatment with PGE (0, 10, 30, 50, 100, 300, and 500 μg/mL) and/or CP (1600 μg/mL) and incubated for 24 h under 5% CO_2_. **a**–**d** Level of cytokines (TNF-α, IFN-γ, IL-2, and IL-12) secreted into the culture medium was analyzed using ELISA kits. Bars labeled with different superscripts have significantly different values (*P* < 0.05). Data are presented as the means ± standard errors (*n* = 3)

To confirm the effect of PGE on production of cytokines by splenocytes, cells were incubated with CP (1600 μg/mL) and PGE (0, 10, 30, 50, 100, 300, or 500 μg/mL) for 24 h. PGE increased cytokine levels in a dose-dependent manner (Fig. 3), which confirmed that PGE attenuates the CP-induced reduction in TNF-α, IFN-γ, IL-2 and IL-12 production by splenocytes.

### Effects of PGE on IgG and IgA levels in CP-treated splenocytes

**Fig. 4:**
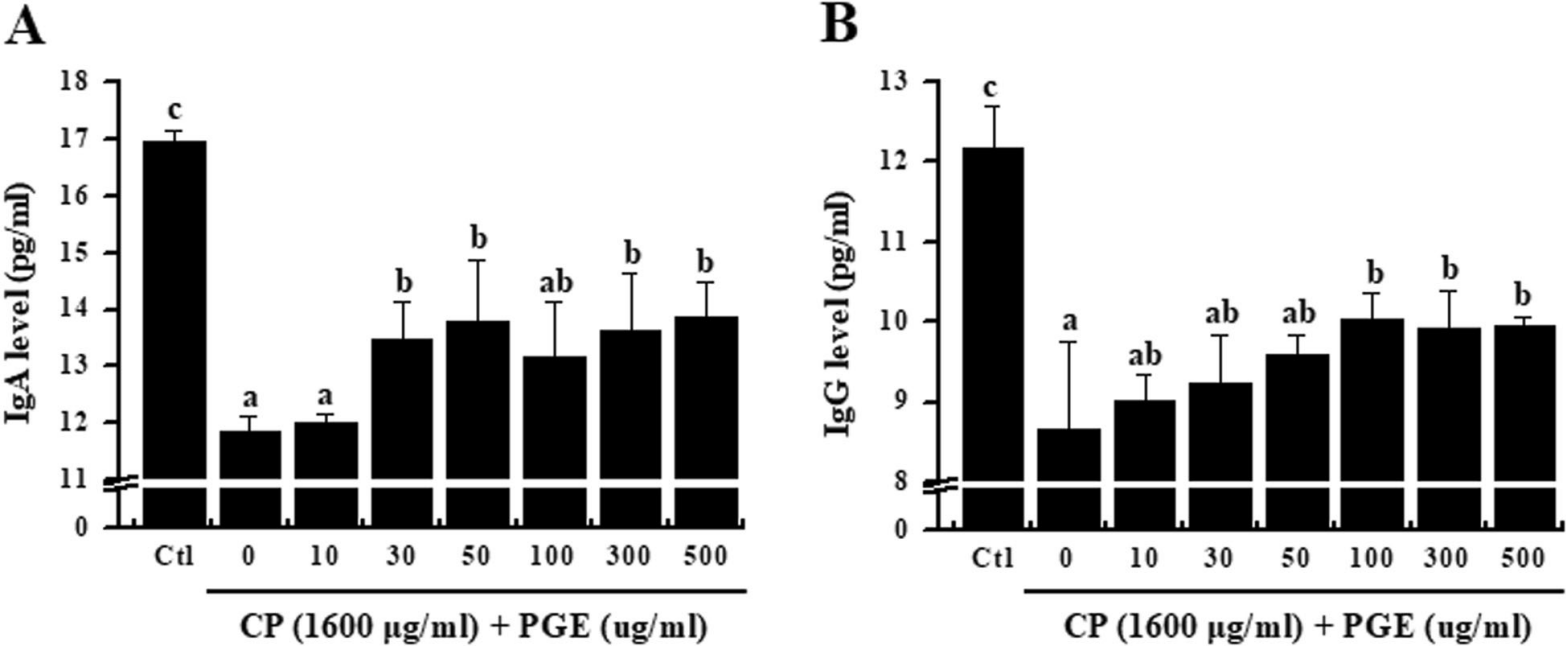
Effect of PGE on immunoglobulin levels in CP-treated splenocytes. Cells were seeded into 96-well plates, followed by treatment with PGE (0, 10, 30, 50, 100, 300, and 500 μg/mL) and/or CP (1600 μg/mL) and incubated for 24 h in a 5% CO2 incubator. Levels of IgG (**a**) and IgA (**b**) secretion into the culture medium were analyzed using ELISA kits. Bars labeled with different superscripts have significantly different values (*P* < 0.05). Data are presented as the means ± standard errors (*n* = 3)

To understand the mechanism underlying the immunoregulatory activity of PGE, levels of IgG and IgA in CP-treated splenocytes were determined by ELISA. As shown in Fig. 4, IgG and IgA levels were significantly decreased upon CP-treatment. However, PGE-treated splenocytes exhibited increased levels of IgG and IgA compared with non-PGE-treated cells, suggesting that PGE improves the humoral immune response.

### Effects of PGE on NK and CTL cytotoxicity in splenocytes

**Fig. 5:**
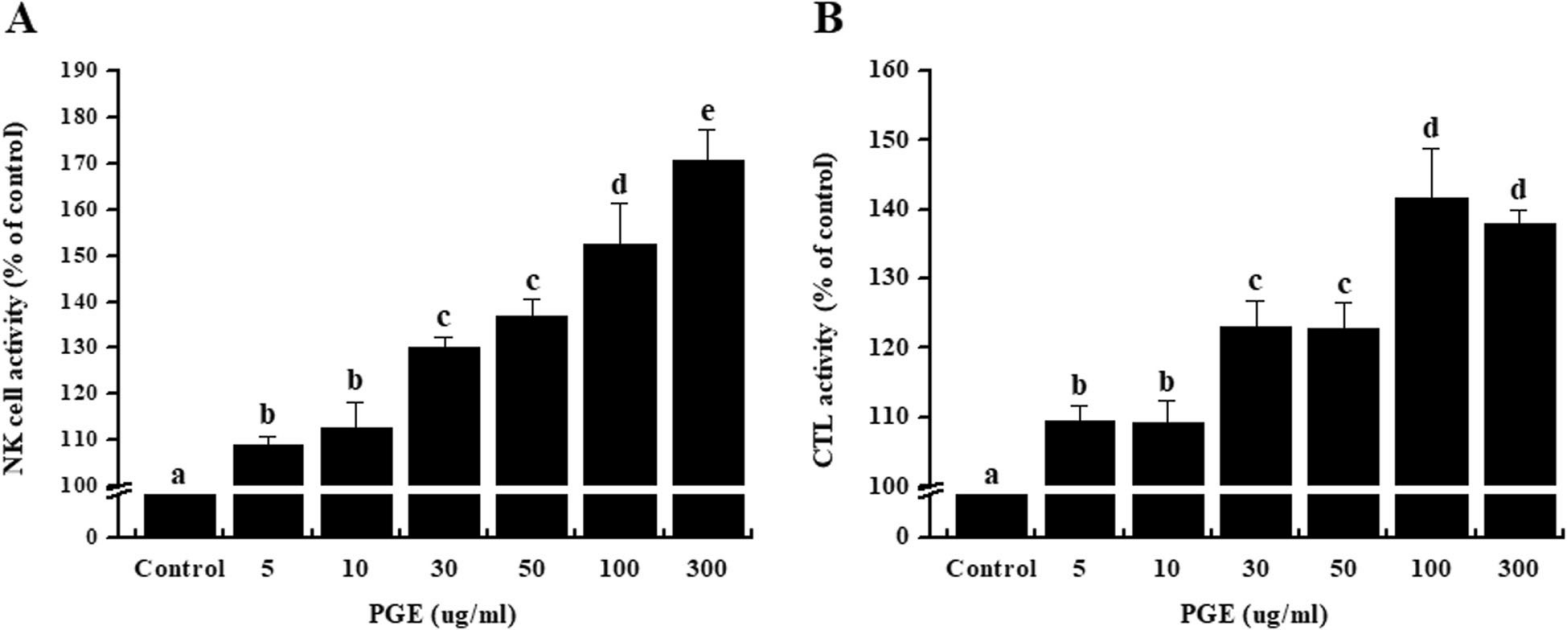
Effect of PGE on splenic NK cell and CTL cytotoxicity in splenocytes. Splenocytes were prepared and assayed for NK cell (**a**) and CTL (**b**) activity using the WST-1 assay. Splenocytes were co-cultured with target cells (AR42J or HL-60) for NK cell or CTL activity, respectively, in 96-well plates, followed by treatment with PGE (0, 5, 10, 30, 50, 100, and 300 μg/mL) and incubated for 24 h in a 5% CO_2_ incubator with an effector to target cell ratio of 25:1. NK cell and CTL activities were calculated as the survival rate of AR42J or HL-60 cells compared to that of control cells. Bars labeled with different superscripts have significantly different values (*P* < 0.05). Data are presented as the means ± standard error (*n* = 3)

NK cells and CTLs are cytotoxic lymphocytes [21] that mediate the defense against tumor cells and virus-infected cells. Therefore, we examined the effects of PGE on NK cell and CTL activity. Splenocyte cytotoxicity was tested against NK-sensitive tumor cells (AR42J) and CTL-sensitive HL-60 cells. As shown in Fig. 5, NK cell and CTL activity were significantly increased after exposure to PGE in a dose-dependent manner, suggesting that PGE can improve the cell immune function in CP-immunosuppressed rats.

### Effect of PGE on immune cell number in rats

**Fig. 6:**
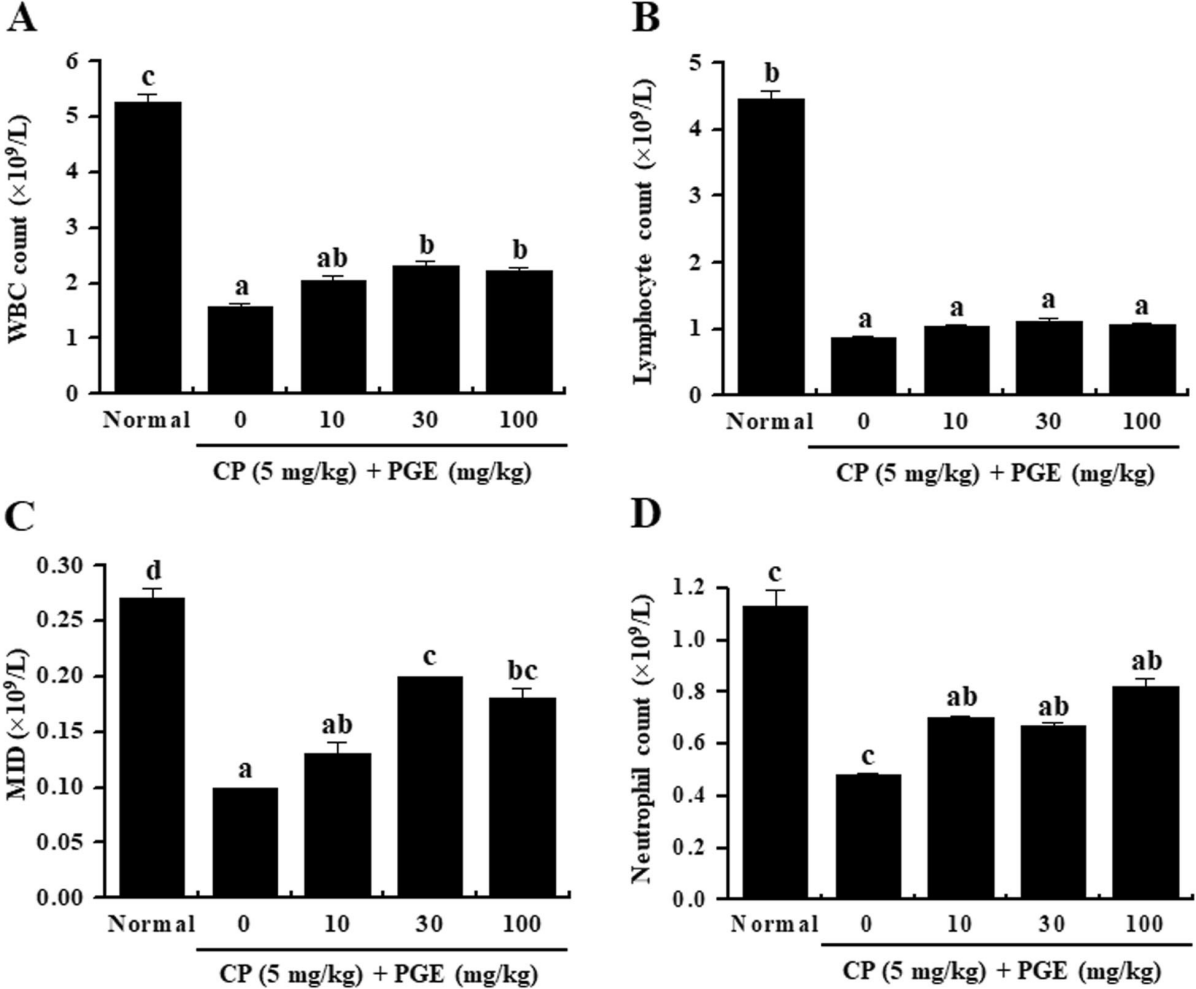
Effects of PGE on inflammatory cell counts in the whole blood of CP-induced immunosuppressed rats. Rats were given saline, CP (5 mg/kg/day), and PGE (0, 10, 30, or 100 mg/kg/day) once daily for 28 days, after which whole blood samples were collected for analysis. **a**–**d** The levels of inflammatory cells (WBCs, lymphocytes, MID, and neutrophils) in the blood samples were determined using a Hemavet 950 system. Bars labeled with different superscripts have significantly different values (*P* < 0.05). Data are presented as the means ± standard errors (*n* = 7)

The immune response is mediated by immune cells, such as T and B lymphocytes, mononuclear cells, and macrophages; thus, these cells play an important role in immune regulation [22–24]. As immunosuppressants such as cyclophosphamide can be used to reduce the population of immune cells, we analyzed the effect of PGE on the cytokine level in immunosuppressed rats that were generated through CP administration. Our results showed that PGE treatment increased the number of WBCs, lymphocytes, MID, and neutrophils in CP-treated rats (Fig. 6).

### Effect of PGE on serum levels of immune-related cytokines in CP-induced immunosuppressed rats

**Fig. 7:**
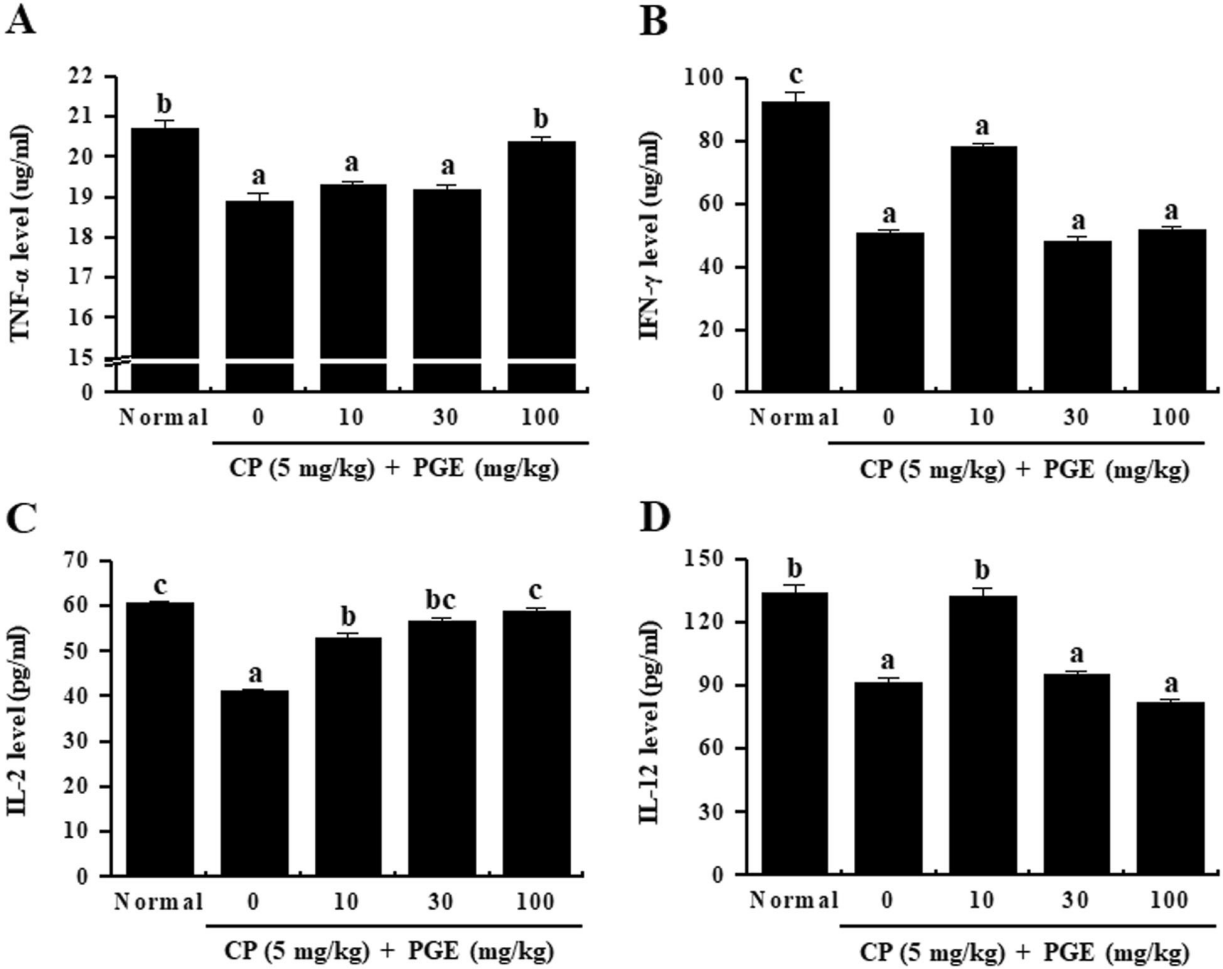
Effect of PGE on the plasma levels of immune-related cytokines in the sera of CP-induced immunosuppression rats. Rats were given saline, CP (5 mg/kg/day), and PGE (0, 10, 30, or 100 mg/kg/day) once daily for 28 days, after which the serum levels of TNF-α (**a**), IFN-γ (**b**), IL-2 (**c**), and IL-12 (**d**) were quantified using ELISA kits. Bars labeled with different superscripts have significantly different values (*P* < 0.05). Data are presented as the means ± standard errors (*n* = 7)

To confirm the effects of PGE on immune-related cytokine expression, we investigated the levels of TNF-α, IFN-γ, IL-2, and IL-12 in the serum of immunosuppressed rats treated with PGE and/or CP (Fig. 7). The level of each of these cytokines was reduced in the serum in immunosuppressed rats (CP-treated) as compared to normal rats (saline-treated). In addition, the PGE-treated group showed a significant increase in the plasma levels of TNF-α and IL-2, compared with the non-PGE treated group, whereas IFN-γ and IL-12 levels showed attenuation in reduction in expression after being upregulated at 10 mg/kg PGE treatment. These results indicate that PGE alleviates the immunosuppression induced by CP treatment by restoring the cytokine production capacity.

### Effects of PGE on serum levels of IgG and IgA in CP-induced immunosuppressed rats

**Fig. 8:**
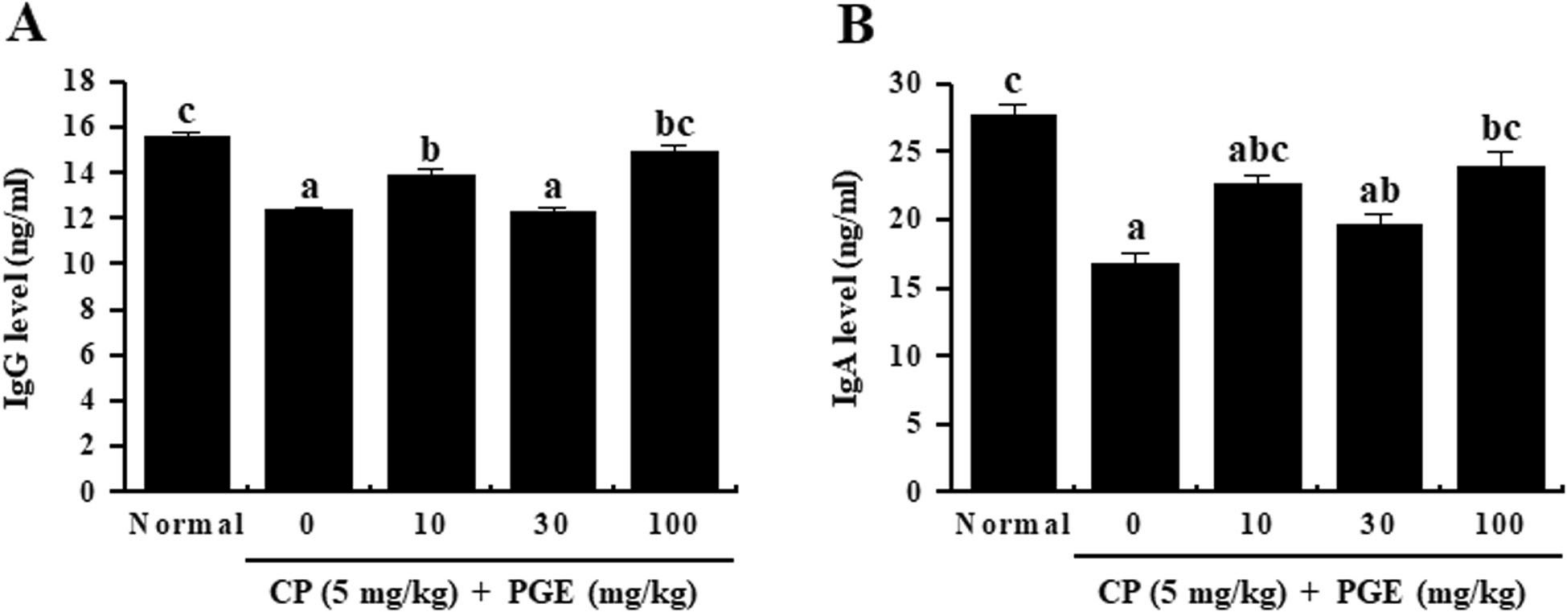
Effect of PGE on plasma levels of immunoglobulins in the sera of CP-induced immunosuppressed rats. Rats were given saline, CP (5 mg/kg/day), and PGE (0, 10, 30, or 100 mg/kg/day) once daily for 28 days, after which the serum levels of IgG (**a**) and IgA (**b**) were quantified using ELISA kits. Bars labeled with different superscripts have significantly different values (*P* < 0.05). Data are presented as the means ± standard errors (*n* = 7)

To understand the mechanism underlying PGE immunoregulatory activity, serum IgG and IgA levels in immunosuppressed rats (CP-treated) were determined by ELISA. As shown in Fig. 8, serum IgG and IgA levels were significantly decreased in the background of CP-induced immunosuppression. However, PGE-treated rats exhibited increased serum IgG and IgA levels compared to non-PGE-treated rats, further confirming that PGE improved the humoral immune response in immunosuppressed rats.

### Effect of PGE on spleen and thymus damage in CP-induced immunosuppressed rats

**Fig. 9:**
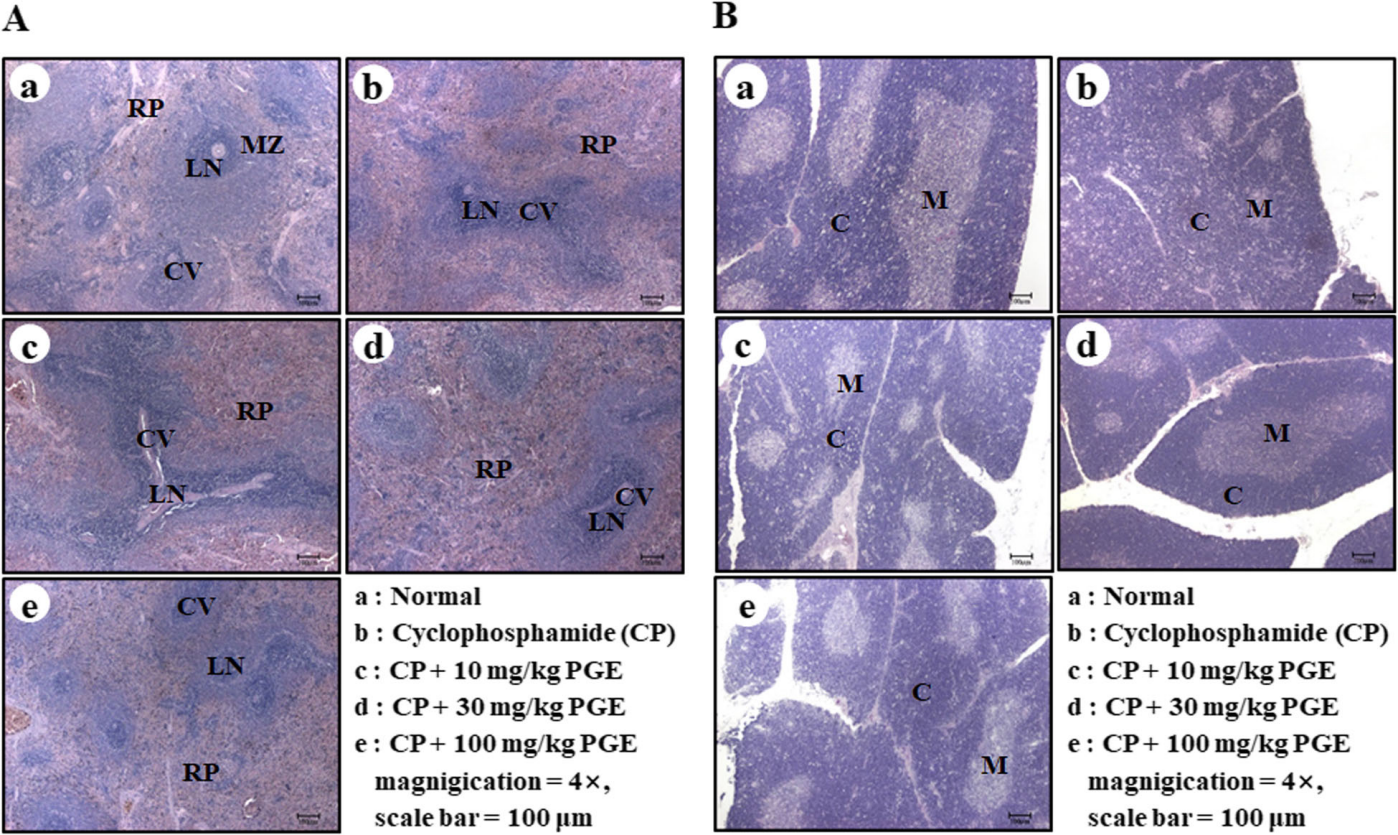
Effect of PGE on immunity-associated spleen and thymus damage in CP-induced immunosuppressed rats. Rats were given saline, CP (5 mg/kg/day), and PGE (0, 10, 30, or 100 mg/kg/day) once daily for 28 days, after which damage in the spleen (**a**) and thymus (**b**) was analyzed histologically. Representative images of the sectioned spleens of (**a**) normal rats (saline treatment), (**b**) control rats (treated with only CP), and (**c**–**e**) CP and PGE-treated rats [(**c**) 10 mg/kg, (**d**) 30 mg/kg, or (**e**) 100 mg/kg PGE]. Scale bar = 100 μm. CV, central vein; LN, lymph nodule; MZ, marginal zone; RP, red pulp; C, cortex; M, medulla

We examined the morphological changes in the spleen and thymus, and also evaluated the effects of PGE treatment on these immune organs. Hematoxylin and eosin staining of the spleen showed that 30 and 100 mg/kg of PGE facilitated spleen cell multiplication and increased the percentage of white pulp, compared to the control group (Fig. 9a). In the thymus, the PGE-treated group exhibited a reduction of the phenomenon whereas the thymus cells, which showed deformation, nuclear condensation, and fragmentation, were separated from the surrounding tissues in a multifocal manner in the cortex (Fig. 9b). Together, these results indicate that PGE stimulates innate and adaptive immunity by restoring the histopathology of the spleen and thymus structures that were damaged by CP treatment.

## Discussion

The study of immune-enhancing effects of natural products represents an active area of current research [18, 25, 26]. Previous studies have reported immune enhancement following administration of PG [27, 28]. However, these reports have only determined the effect of PG on immune enhancement of lymphocytes and macrophages. In this study, we first showed that PG could revert the immunosuppression caused by CP. Specifically, PGE-treatment alleviated CP-induced reduction in cell proliferation, cytokine and immunoglobulin levels, and NK cell and CTL activity in splenocytes. Additionally, PGE increased immune cell numbers and serum cytokines and immunoglobulin levels, and stimulated the recovery of the spleen and thymus in immunosuppressed rats. These findings indicate that PGE improves the weakened immune response brought about by CP treatment.

CP is an important chemotherapeutic drug in tumor therapy, but it is not beneficial for healthy cells and side effects such as bone marrow suppression, immunosuppression, oxidative stress, and occasionally life-threatening effects, are often observed [29]. In previous studies, CP-treated rats have been used as immunosuppression models in animals. CP-induced immunosuppression significantly reduces red blood cell, WBC, and platelet counts, and additionally inhibits spleen NK cell and CTL activity, as well as decreasing CD4 T lymphocyte count and the CD4/CD8 ratio [30, 31]. In addition, the levels of cytokines (IL-2, IFN, and IL-10) and immunoglobulins (IgA, IgM, and IgG), along with serum hemoglobin, were reduced by CP [32]. Moreover, administration of CP has been shown to cause damage to the spleen and thymus, which are important organs in the immune response [33]. Thus, we investigated the immune-enhancing effects of PGE under conditions of CP-induced immunosuppression in this study.

Proliferation of macrophages and lymphocytes is important in the early activation stage of cellular and humoral immune responses. Splenocytes are composed of various cells, such as T and B cells, macrophages, and dendritic cells with different immune functions [34]. Stimulation of splenocytes proliferation ultimately increases the expression of cytokines, potentially improving cell-mediated immune responses and leading to immune-enhancement [35]. We found that PGE reversed the effect of reduced immune cell viability caused by CP (Fig. 2b and Fig. 6).

Cytokines expressed by various immune cells play an important role in lymphocyte differentiation, inflammation control, host defenses against bacterial infection, cell survival, apoptosis, and the immune response [36, 37]. Th cells activated after antigen recognition are classified into Th1 and Th2 cells based on their function and differences in cytokines secreted [38, 39] . The different types of cytokines secreted by Th1 and Th2 cells are important factors in determining cell function. Th1 cells secrete IL-2, TNF-α, and IFN-γ, which are involved in cell-mediated immune responses [40, 41], whereas Th2 cells mainly secrete IL-4, IL-6, and IL-10 through the humoral immune response [42]. In particular, TNF-α is produced by T and B cells, NK cells, and macrophages, and regulates inflammation and the host defense through inhibiting bacterial infections and suppressing acute stress [43, 44]. IFN-γ and IL-2 are produced by T-helper cells [45]. IFN-γ is a natural immune mediator that promotes the expression of major histocompatibility complex molecules through activation of monocytes/macrophages [46]. IL-2, produced by activated T lymphocytes, induces growth, proliferation, and differentiation of immature T cells into effector T cells [47]. Finally, IL-12 is known to be produced by a variety of immune cells, including dendritic cells, macrophages, neutrophils, and human B-lymphocytes. It is responsible for differentiating native T lymphocytes into Th1 lymphocytes [48], and promotes the production of TNF-α and IFN-γ in T lymphocytes and NK cells, respectively [49, 50]. Therefore, we confirmed the effect of PGE on the expression of Th1 cytokines, and hypothesized that PGE would promote the Th1 immune response under conditions of CP-induced immunosuppression (Fig. 3 and Fig. 7).

IgG, IgA, and IgM comprise major immunoglobulins that play a role in the neutralization of toxins, bacteria, or viruses, along with opsonization and complement activation [51]. Recently, numerous studies have reported that natural products can enhance the humoral immune response by promoting the production of IgA, IgM, and IgG [16]. Our results demonstrate that PGE can increase levels of IgG and IgA in CP-immunosuppressed rats. This result suggests that PGE plays a role in enhancing the humoral immune response.

NK cells and CTLs comprise two major populations that can target and kill foreign and abnormal cells. NK cells and CTLs play an important role in the early immune response [52]. NK cells and CTLs are activated by stimulation of cytokines and chemokines and play a central role in the regulation of tumor growth and metastasis, as well as the removal of viruses [52]. Thus, measurement of NK cell and CTL activity represents a useful method for assessing the cellular immune response of the host [53]. Our results showed that spleen NK cell cytotoxic activity was significantly increased after treatment with PGE (Fig. 5). Additionally, we assessed the NK cell and CTL activity of Red ginseng extract (RGE) as a positive control, and found that, although the RGE extracts were highly cytotoxic, NK cell activity was greater in the RGE group than in the PGE group at concentrations below 50 μg/mL. However, CTL activity was higher in the PGE group compared to the RGE group, and in particular, PGE had a pronounced effect on CTL activity, which was higher than for the RGE group (Additional file 1: Figure S1). These results suggest that PGE enhances the cell-mediated immune response through modulating NK cell activity.

Immunosuppression is a condition in which the immune system is not sufficiently activated to defend the host from infections. The immune system can be weakened by various factors, such as birth and aging, which may lead to shrinkage of immune tissues and a decrease in the number of WBCs [54]. Mature blood cells such as WBCs, red blood cells, and platelets are produced by hematopoietic stem cells, which are multipotent and self-renewable [55]. We found that, although CP treatment decreased the WBC, lymphocyte, MID, and neutrophil populations, consistent with prior reports [56], these cell counts were significantly restored by PGE administration in a dose-dependent manner. These results suggest that PGE enhances immunity by protecting against CP-induced myelosuppression.

In addition, we confirmed the effect of PGE on CP-induced immune organ damage. Histopathological examination of the spleen and thymus of immunosuppressed rats (CP-treated) revealed CP-induced damage. Notably, PGE-treatment significantly reduced CP-induced pathological changes in the spleen and thymus (Fig. 9), suggesting that PGE may in fact reverse the CP-induced atrophy in immune organs.

## Conclusion

In summary, we demonstrated the immune-enhancing effects of PGE in CP-treated splenocytes using an immunosuppressed rat model. Our results confirmed that PGE stimulates innate and adaptive immune responses under immunosuppressive conditions, and increases cytokine, antibody, and immune cell production and NK cell and CTL activity, thereby enhancing the immune response and host defense. These findings suggest that PG may have applications in the development of functional foods and medicines that can be used for immune-enhancement.

## Supplementary information

**Additional file 1: Figure S1.** Effect of PGE compared to RGE on splenic NK cell activity in splenocytes. (A) Splenocytes were seeded into a 96-well plate with RGE (0, 3, 5, 10, 30, 50, 100, 300, 500, 1000 and 2000 μg/mL). Cells were incubated for 24 h under 5% CO_2_, and their viability was measured using a WST-1 Assay Kit. (B, C) Splenocytes were co-cultured with AR42J or HL-60 target cells to induce NK cell or CTL activity, respectively, in 96-well plates, followed by treatment with RGE (0, 5, 10, 30, 50, 100, and 300 μg/mL) and PGE (0, 5, 10, 30, 50, 100, and 300 μg/mL). Cells were then incubated for 24 h in a 5% CO_2_ incubator with an effector to target cells ratio of 25:1. NK cell and CTL activities were calculated as the survival rate of AR42J or HL-60 cells compared to that of control cells. Bars labeled with different superscripts have significantly different values (*P* < 0.05). Data are presented as the means ± standard errors (*n* = 3).

## Data Availability

The datasets analyzed during the current study are available from the corresponding author on reasonable request.

## Abbreviations

CBC: Complete blood count
CP: Cyclophosphamide
CTL: Cytotoxic T lymphocyte
MID: Mid-range absolute counts
NK: Natural killer
PGE: *P. grandiflorum* extract
