# Supplementary figures and images for: Immuno-enhancement effects of Platycodon grandiflorum extracts in splenocytes and a cyclophosphamide-induced immunosuppressed rat model
Source: BMC Complement Altern Med. 2019 Nov 21;19:322. doi: 10.1186/s12906-019-2724-0 (PMC6868875; doi:10.1186/s12906-019-2724-0)

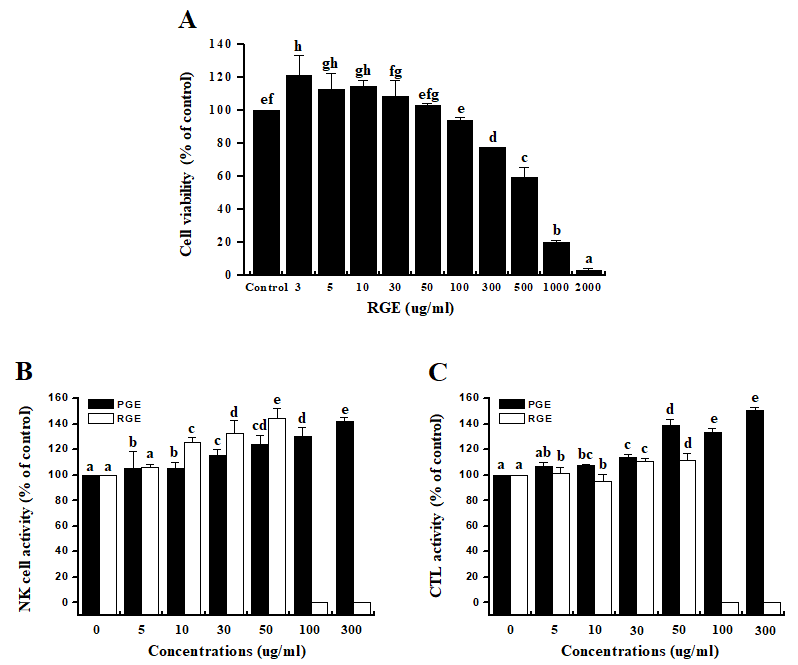

Supplement: Supplementary file 1 — Additional file 1: Figure S1. Effect of PGE compared to RGE on splenic NK cell activity in splenocytes. (A) Splenocytes were seeded into a 96-well plate with RGE (0, 3, 5, 10, 30, 50, 100, 300, 500, 1000 and 2000 μg/mL). Cells were incubated for 24 h under 5% CO2, and their viability was measured using a WST-1 Assay Kit. (B, C) Splenocytes were co-cultured with AR42J or HL-60 target cells to induce NK cell or CTL activity, respectively, in 96-well plates, followed by treatment with RGE (0, 5, 10, 30, 50, 100, and 300 μg/mL) and PGE (0, 5, 10, 30, 50, 100, and 300 μg/mL). Cells were then incubated for 24 h in a 5% CO2 incubator with an effector to target cells ratio of 25:1. NK cell and CTL activities were calculated as the survival rate of AR42J or HL-60 cells compared to that of control cells. Bars labeled with different superscripts have significantly different values (P < 0.05). Data are presented as the means ± standard errors (n = 3). [file 12906_2019_2724_MOESM1_ESM.tif]
